# Computed tomographic assessment of lung aeration at different positive end-expiratory pressures in a porcine model of intra-abdominal hypertension and lung injury

**DOI:** 10.1186/s40635-021-00416-5

**Published:** 2021-10-04

**Authors:** Adrian Regli, Siavash Ahmadi-Noorbakhsh, Gabrielle Christine Musk, David Joseph Reese, Peter Herrmann, Martin Joseph Firth, J. Jane Pillow

**Affiliations:** 1 Department of Intensive Care, Fiona Stanley Hospital, Murdoch Drive, Murdoch, WA 6150 Australia; 2 Medical School, Division of Emergency Medicine, The University of Western Australia, 35 Stirling Highway, Crawley, 6009 Australia; 3 Medical School, The University of Notre Dame Australia, 19 Mouat Street, Fremantle, 6959 Australia; 4 School of Human Sciences, The University of Western Australia, 35 Stirling Highway, Crawley, 6009 Australia; 5 Animal Care Services, The University of Western Australia, 35 Stirling Highway, Crawley, 6009 Australia; 6 School of Veterinary and Life Sciences, Murdoch University, Nyarrie Drive, Murdoch, 6150 Australia; 7 VetCT Consultants in Telemedicine PTY LTD, 185-187 High Street, Fremantle, 6160 Australia; 8 Department of Anaesthesiology, Emergency and Intensive Care Medicine, University of Göttingen, Robert-Koch-Str. 40, 37075 Göttingen, Germany; 9 Centre for Applied Statistics, Department of Mathematics and Statistics, The University of Western Australia, 35 Stirling Highway, Crawley, 6009 Australia

**Keywords:** Intra-abdominal hypertension, Positive end-expiratory pressure, Computed tomography, Lung volumes, Acute lung injury, Abdominal compartment syndrome, Over-distension, Atelectasis, Mechanical ventilation

## Abstract

**Background:**

Intra-abdominal hypertension (IAH) is common in critically ill patients and is associated with increased morbidity and mortality. High positive end-expiratory pressures (PEEP) can reverse lung volume and oxygenation decline caused by IAH, but its impact on alveolar overdistension is less clear. We aimed to find a PEEP range that would be high enough to reduce atelectasis, while low enough to minimize alveolar overdistention in the presence of IAH and lung injury.

**Methods:**

Five anesthetized pigs received standardized anesthesia and mechanical ventilation. Peritoneal insufflation of air was used to generate intra-abdominal pressure of 27 cmH_2_O. Lung injury was created by intravenous oleic acid. PEEP levels of 5, 12, 17, 22, and 27 cmH_2_O were applied. We performed computed tomography and measured arterial oxygen levels, respiratory mechanics, and cardiac output 5 min after each new PEEP level. The proportion of overdistended, normally aerated, poorly aerated, and non-aerated atelectatic lung tissue was calculated based on Hounsfield units.

**Results:**

PEEP decreased the proportion of poorly aerated and atelectatic lung, while increasing normally aerated lung. Overdistension increased with each incremental increase in applied PEEP. “Best PEEP” (respiratory mechanics or oxygenation) was higher than the “optimal CT inflation PEEP range” (difference between lower inflection points of atelectatic and overdistended lung) in healthy and injured lungs.

**Conclusions:**

Our findings in a large animal model suggest that titrating a PEEP to respiratory mechanics or oxygenation in the presence of IAH is associated with increased alveolar overdistension.

**Supplementary Information:**

The online version contains supplementary material available at 10.1186/s40635-021-00416-5.

## Background

Intra-abdominal hypertension (IAH) is defined as a sustained intra-abdominal pressure (IAP) above or equal to 12 mmHg [1] and occurs in around 30% of critically ill patients. Mortality increases in proportion to the degree of IAH [2].

IAH impairs function and causes permanent histological changes of various organ systems [3–5]. Furthermore, due to a cephalad shift of the diaphragm, IAH causes pulmonary atelectasis, impaired lung function and chest wall compliance as well as reduced oxygenation [4, 6–14].

Subsequently, patients with IAH frequently require mechanical ventilation, not only because IAH impairs lung function, but also as measures to reduce IAP can be ineffective [15, 16]. However, the optimal mechanical ventilation, and more specifically, the optimal level of PEEP in patients with IAH remains debated [7, 13, 14, 17–19].

Previous experimental results show that high PEEP levels counteract the adverse respiratory effects of IAH, including lung volumes, respiratory mechanics and oxygenation [8, 11, 13, 20, 21]. Furthermore, higher PEEP levels may reduce the risk of ventilation-induced lung injury by preventing cyclic collapsing and reopening of alveoli in the dependent lung regions of patients with IAH [22, 23].

However, high PEEP levels might not only compromise the cardiovascular system but also cause alveolar over-distension in the non-dependent lung regions, which is associated with ventilator-induced lung injury [17–19, 22, 24].

Therefore, this study aimed to investigate the effect of PEEP on the degree of overdistension using computed tomography (CT) [25, 26]. We set out to find an “optimal CT inflation PEEP range” that would be high enough to reduce atelectasis formation while causing minimal overdistention in a pig model of IAH and lung injury.

## Methods

Additional details of the study methodology are provided in the online supplement (Additional file 1: 1. methods unabridged).

The study conformed to the regulations of the Australian Code for the care and use of animals for scientific purposes [27] and was approved by the Animal Ethics Committees, Murdoch University (R2588/13).

### Preparation of animals and ventilation

Five anesthetized and paralyzed female pigs (Large White) with a median (IQR) weight of 29.3 (29.0–30.6) kg were included in this study.

The pigs were mechanically ventilated (Babylog VN500, Draeger, Lübeck, Germany) using the following settings: volume guaranteed pressure-controlled continuous mandatory ventilation (PC-CMV/VG), F_i_O_2_ 0.6, tidal volume 8 mL/kg. The initial PEEP setting was 5 cmH_2_O and altered according to the experimental protocol (see below). The initial respiratory rate was adjusted to maintain an end-tidal CO_2_ of 35–45 mmHg. Subsequently, PEEP was the only ventilation setting altered throughout the remainder of the protocol.

### Lung injury

The experimental protocol was carried out first with healthy lungs and then with injured lungs (Additional file 1: 2. Figure: Experimental process). To create lung injury, we used oleic acid as previously described [11] until a P/F ratio of < 300 mmHg was established. Mild as opposed to moderate or severe lung injury was chosen due to safety concerns in the setting of IAH.

### Respiratory mechanics

End-inspiratory and end-expiratory airway and esophageal pressures were obtained and the static elastances of the respiratory system (*E*_rs_), chest wall (*E*_*W*_) and lung (*E*_*L*_) and the transpulmonary pressures were derived as described previously [11]. Arterial oxygen and carbon dioxide tension were measured and PaO_2_ over fractional inspiratory oxygen concentration (*P*/*F* ratio) was calculated [11].

### Hemodynamic parameters

The animals remained supine throughout the study. Mean arterial blood pressure was measured at the femoral artery and cardiac output was measured by transpulmonary thermodilution [11].

Pigs were stabilized hemodynamically with 4% succinylated gelatin solution (500 mL over the first 30 min followed by 1 mL/kg/h, Gelofusine®, Braun, Bella Vista NSW, Australia). Noradrenalin infusion (3 mg/50 mL) was administered if required to maintain a mean arterial pressure ≥ 70 mmHg.

### Intra-abdominal pressure

A large bore orogastric tube was inserted to allow continuous gastric drainage. IAP of 27 cmH_2_O (20 mmHg) was created by the insufflation of air into the peritoneal cavity through an air-tight catheter. IAP ≥ 27 cmH_2_O represents ≥ grade III IAH found in around 8% of patients [1, 2, 16]. A three-way tap connected to a transducer allowed direct measurement of IAP [6].

### Experimental protocol

After performing baseline measurements, IAP of 27 cmH_2_O (20 mmHg) was applied. The initial PEEP of 5 cmH_2_O was first incrementally increased (“ascending”) and then decreased (“descending”). The following increments/decrements of PEEP were applied: 5, 12, 17, 22, and 27 cmH_2_O. Recruitment maneuvers were not used. Ventilation settings were kept constant except for PEEP. All measurements were obtained 5 min after a stabilization period; physiological measurements at each PEEP level and CT measurements (see below) were only obtained at descending PEEP levels. Hysteresis was assessed by comparing measurements obtained at ascending and descending PEEP levels.

### Computed tomography

A whole-lung helical CT scan (Siemens Somatom Emotion 16, Erlangen, Germany) was performed during an inspiratory and an expiratory pause (each about 20 s) [28]. The scan parameters were standardized to 130 kV, 110 mA, 1.0 pitch, and 3 mm slice thickness at each tested PEEP level.

Image analyses was performed with Maluna® software (MALUNA 3.17, Peter Herrmann, University of Göttingen, Göttingen, Germany) [6]. Based on the lung tissue density, four aeration compartments were computed: overdistended (− 1000 to − 901 Hounsfield units [HU]), normally aerated (− 900 to − 501 HU), poorly aerated (− 500 to − 101 HU), and non-aerated (atelectatic) lung tissue (− 100 to 200 HU) [6, 29]. Lung volumes were calculated for three segments along the dorso-ventral axis.

CT lung volumes were further analyzed in Excel (v16 for Mac, Microsoft, Redmond, Washington, USA) to derive equations that best-fitted pressure (PEEP levels) – volume (CT volumes) curves for each aeration compartment separately. Three different equations, including the Venegas equation (*V* = *a* + [*b*/(1 + *e*^–(*P*–*c*)/*d*^)]), were assessed [25, 30, 31]. The best fit defined the curve resulting in the smallest root mean square between the measured and calculated pressure–volume points. We calculated lower and upper inflection points as previously described [30]. We defined an “optimal CT inflation PEEP range” as the difference between the lower inflection points (*P* = *c* − 2*d*) of both the atelectatic and overdistended lung.

### Statistics

A linear mixed model was applied to assess the effect of factors (IAH, lung injury, ascending vs descending PEEP) and covariates (PEEP) on different variables using SPSS (v25, IBM, St Leonards NSW, Australia). This approach accounted for the correlation between the repeated measures on each pig. Main effect was used for analysis of respiratory and hemodynamic outcomes. Main effect plus an interaction with lung segments (PEEP and lung injury) was used for CT measured lung volumes. Laterality (left/right) was included as a fixed factor in the linear mixed model. Differences between pigs were accounted for as a random effect. Missing values were imputed based on the average relative differences between any pig with missing data and the other animals. Wilcoxon-Signed Rank test was used to compare optimum PEEP levels between healthy and injured lungs. Linear regression was performed to assess for correlations. A *p* value of < 0.05 was considered statistically significant. For descriptive statistics, median (IQR) is reported.

## Results

One pig died during the protocol after lung injury was induced at the highest PEEP level of 27 cmH_2_O. Therefore, we were unable to perform CT analysis or cardio-respiratory measurements with descending PEEP levels in this pig. The remaining results were used as described above. All other pigs survived to study completion.

### Effect of oleic acid

To create lung injury, we required 0.4 (0.4–0.8) mL/kg IV oleic acid. The resulting P/F ratio of injured lungs before abdominal inflation was 153 (146–232) mmHg (Table 1, Additional file 1: 3. Figure: Effect of IAH and lung injury on P/F ratio). Lung injury increased plateau airway and expiratory esophageal pressure, and increased *E*_rs_ consequent to increased *E*_*L*_.

**Table 1:** Effect of PEEP on expiratory cardio-respiratory variables

IAP, cmH_2_O	BL	27	27	27	27	27	P, PEEP
PEEP, cmH_2_O (% of IAP)	5	5 (19)	12 (44)	17 (63)	22 (81)	27 (100)	
**Healthy lungs**							
*P*/*F* ratio, mmHg	542 (539,547)	502 (499,504)	**532 (492,554)**	512 (512,549)	513 (502,522)	515 (514,546)	0.37
*P*_aw, insp_, cmH_2_O	17 (16,17)	31 (31,33)^#^	37 (36,38)	41 (38,41)	43 (42,44)	51 (51,52)	** < 0.01**
*P*_es, exp_, cmH_2_O	10 (6,11)	12 (9,12)	17 (14,18)	21 (19,21)	24 (23,25)	27 (26,27)	** < 0.01**
*P*_es, insp_, cmH_2_O	13 (11,14)	29 (28,30)^#^	34 (31,34)	36 (33,36)	38 (36,38)	43(41,43)	** < 0.01**
*E*_rs_, cmH_2_O/L	39 (39,42)	103 (99,103)^#^	94 (93,99)	85 (84,86)	**81 (75,82)**	92 (87,100)	< 0.01 *
*E*_W_, cmH_2_O/L	16 (14,18)	67 (64,73)^#^	66 (59,71)	**53 (53,62)**	55 (53,56)	60 (60,61)	0.71
*E*_L_, cmH_2_O/L	26 (21,28)	35 (30,41)^#^	35 (27,35)	30 (25,32)	**27 (25,27)**	31 (27,33)	**0.02**
C.O., L/min	4.2 (4.1,4.5)	**4.4 (4.0,4.7)**	3.9 (3.6,3.9)	3.2 (3.2,3.4)	2.9 (2.8,2.9)	2.8 (2.7,2.9)	** < 0.01**
Total lung volume, L	1.2 (1.1,1.2)	0.8 (0.7,0.8)^#^	0.9 (0.9,1.0)	1.0 (1.0,1.0)	1.1 (1.1,1.2)	1.3 (1.2,1.3)	** < 0.01**
Lung gas volume, mL	721 (698,791)	378 (370,418)^#^	511 (501,561)	620 (611,649)	761 (743,788)	903 (856,908)	< 0.01
Lung tissue mass, g	393 (356,412)	338 (316,354)	355 (334,358)	352 (329,361)	353 (338,369)	351 (322,367)	0.60
Overdistended, %	2 (2,2)	1 (1,2)	2 (1,3)	2 (2,4)	4 (3,5)	5 (4,7)	** < 0.01**
Normally aerated, %	82 (79,85)	56 (50,65)^#^	68 (63,74)	75 (70,80)	79 (76,83)	82 (82,86)	** < 0.01**
Poorly aerated, %	14 (11,16)	32 (27,36)^#^	23 (20,38)	18 (15,22)	14 (10,16)	9 (8,11)	** < 0.01**
Atelectatic, %	2 (1,4)	10 (7,15)	5 (4,7)	3 (2,4)	3 (2,3)	2 (1,2)	** < 0.01**
**Injured lungs**							
*P*/*F* ratio, mmHg	153 (146,232)^‡^	175 (163,201)^‡^	235 (187,272)	244 (178,326)	**285 (240,409)**	243 (165,312)	0.04 *
*P*_aw, insp_, cmH_2_O	23 (23,24)^‡^	38 (31,40)^#‡^	40 (40,41)	44 (42,44)	47 (46,47)	57 (54,57)	< 0.01 *
*P*_es, exp_, cmH_2_O	10 (9,19)	30 (30,32)	34 (32,38)	38 (34,39)	40 (37,41)	44 (39,45)	< 0.01 *
*P*_es, insp_, cmH_2_O	17 (14,23)	16 (13,18)^#^	20 (18,22)	23 (23,24)	26 (26,28)	29 (29,30)	** < 0.01**
*E*_rs,_ cmH_2_O/L	73 (62,75)^‡^	115 (101,148)^#‡^	109 (102,116)	101 (92,103)	**89 (85,95)**	105 (104,106)	< 0.01 *
*E*_W_, cmH_2_O/L	19 (17,19)	62 (56,63)^#^	61 (50,73)	54 (46,59)	**53 (52,53)**	57 (50,62)	0.40
*E*_L_, cmH_2_O/L	50 (4,53)^‡^	53 (49,74)^‡^	42 (40,50)	38 (36,46)	**35 (34,38)**	48 (45,52)	< 0.01 *
C.O., L/min	4.0 (3.4,4.8)	**4.2 (4.0,4.5)**	3.3 (3.0,3.7)	3.2 (2.8,3.2)	3.1 (3.0,3.6)	3.3 (2.5,3.9)	**0.01**
Total lung volume, L	1.0 (1.0,1.0)	1.0 (0.9,1.1)	1.2 (1.0,1.2)	1.3 (1.0,1.4)	1.5 (1.2,1.5)	1.7 (1.3,1.7)	** < 0.01**
Lung gas volume, mL	522 (519,596)^‡^	428 (381,449)	569 (482,609)	744 (570,762)	885 (732,933)	1024 (827,1104)	** < 0.01**
Lung tissue mass, g	397 (372,399)	446 (361,454)	489 (381,521)	526 (392,546)	546 (424,557)	548 (429,560)	**0.01**
Overdistended, %	2 (2,3)	2 (1,2)	2 (2,3)	3 (2,3)	3 (3,3)	5 (4,6)	** < 0.01**
Normally aerated, %	61 (57,66)^‡^	44 (35,51)	55 (45,59)	60 (53,65)	63 (58,70)	69 (65,74)	** < 0.01**
Poorly aerated, %	24 (22,26)	26 (22,31)	25 (22,29)	24 (21,29)	24 (18,27)	19 (15,25)	** < 0.01**
Atelectatic, %	11 (8,13)	26 (15,35)^#‡^	18 (10,23)	11 (7,15)	6 (3,7)	3 (24)	** < 0.01**

Bold indicates significant *p*-values or best values to determine optimum PEEP levels

BL incidcates baseline intra-abdominal pressure (IAP), otherwise IAP of 27 cmH_2_O (20 mmHg) was applied. Positive end-expiratory pressure (PEEP) was increased stepwise (ascending) then decreased (descending). Only descending values are provided. Hysteresis, difference between ascending and descending PEEP; *P*/*F* ratio, arterial oxygen tension/fractional inspiratory concentration of oxygen; *P*_aw_ plateau airway pressure; *P*_es_ esophageal pressure; _insp_ end-inspiratory; _exp_ end-expiratory; *E eslastanc*; _*rs*_ respiratory system; _*W*_ chest wall; _*L*_ lung; *C.O.* cardiac output; *P*_a, mean_ mean arterial pressure. Median (IQR) are given. Mixed linear effects model was used for statistical analysis

^#^*p* < 0.05 between before (BL) and after IAH

^‡^*p* < 0.5 between healthy and injured lungs

*p < 0.05 indicating presence of hysteresis

### Effect of IAH

Median (IQR) baseline IAP were 2 (0–5) cmH_2_O [2 (0–4) mmHg] with healthy lungs and 3 (3–3) cmH_2_O [3 (2–3) mmHg] after oleic acid (lung injury). Overall IAH decreased oxygenation but this finding was not confirmed in a subgroup analyzes of healthy or sick lungs (Table 1, Additional file 1: 3. Figure: Effect of IAH and lung injury on *P*/*F* ratio). Plateau airway and inspiratory esophageal pressures, *E*_*W*_ and *E*_*L*_ increased in the presence of IAH.

### Effect of lung injury and IAH on CT parameters

Because the effect of IAH, oleic acid and PEEP on segmental lung aeration at end-inspiration paralleled those at end-expiration, we only describe lung aeration measured during end-expiration. Inspiratory values are presented in Additional file 1: 4. Table: Effect of PEEP on inspiratory lung aeration. While lung injury decreased gas volumes, IAH decreased gas volumes only in healthy but not in injured lungs (Table 1). Neither lung injury nor IAH affected tissue mass. The effect of lung injury and IAH were more pronounced on a segmental level (Additional file 1: 5. Table: Effect of IAH and lung injury on segmental lung aeration, Additional file 1: 6. Figure: Effect of IAH and lung injury on segmental lung volumes). In the dorsal dependent lung segments, lung injury and IAH decreased the segmental proportion of normally aerated lung due to an increase in atelectatic lung.

**Fig. 1:**
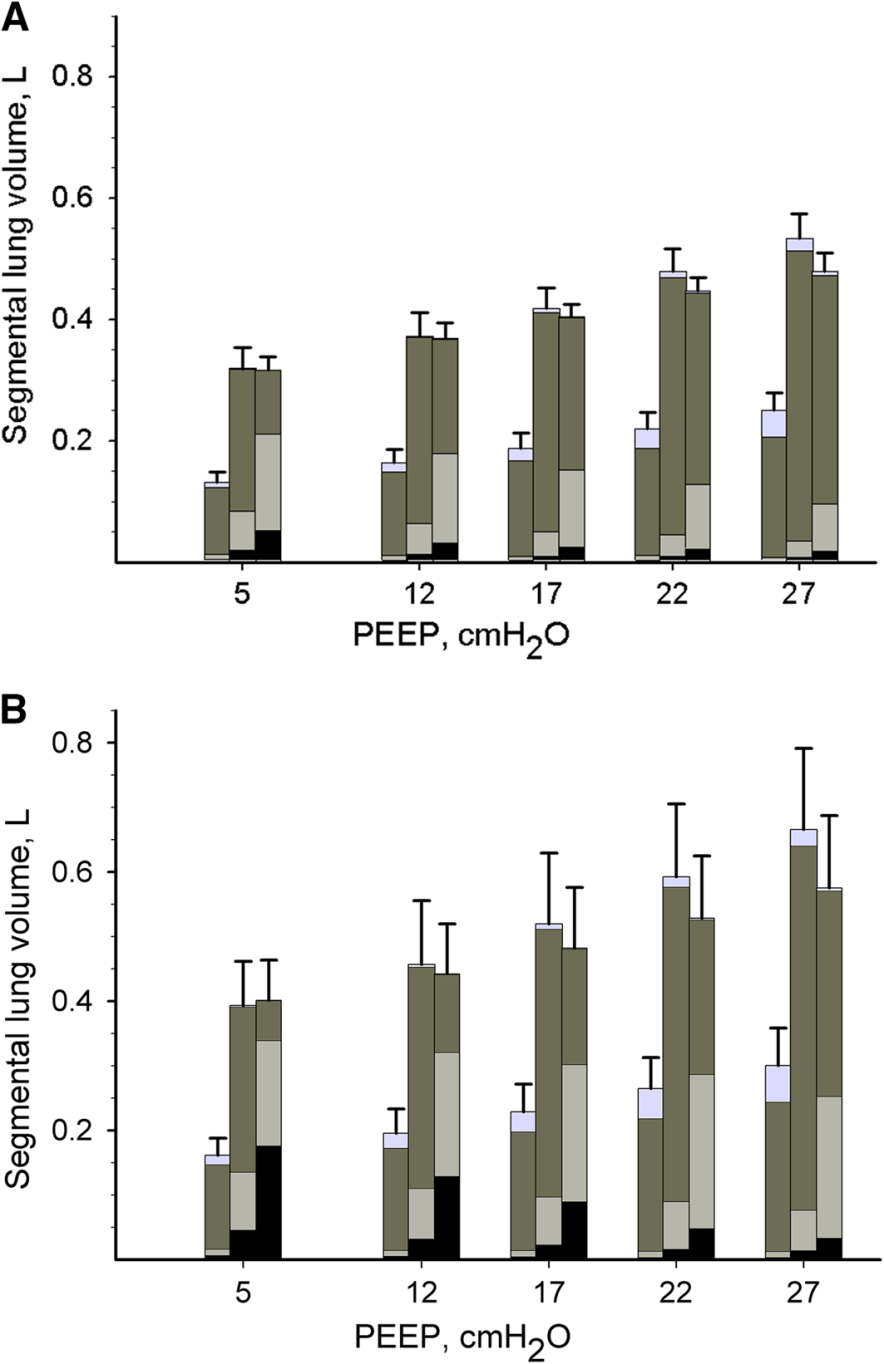
Effect of descending positive end expiratory pressure (PEEP) level on end-expiratory segmental lung volumes measured by computed tomography with healthy lungs (**a**) and after creating lung injury (**b**). Abdomen was inflated to an intra-abdominal pressure of 27 cmH_2_O (20 mmHg). Three lung segments are depicted at each condition with left to right representing ventral, medial and dorsal lung segment, respectively. Segmental lung volumes are a composite of overdistended (light grey, − 1000 to − 901 HU), normally aerated (dark grey, − 900 to − 501 HU), poorly aerated (light grey third from top, − 500 to − 101 HU) and non-aerated atelectatic lung (black, − 100 to 200 HU). Mean and SE are shown. See Table 1 for statistical comparisons

Throughout the different experimental conditions, atelectatic and poorly aerated lung changes dominated in the dorsal dependent lung segments, whereas the changes in overdistension dominated in the ventral non-dependent lung segments (Fig. 1, Additional file 1: 5. Table: Effect of IAH and lung injury on segmental lung aeration, Additional file 1: 7. Table: Effect of PEEP on ventral and dorsal aeration).

The proportion of atelectatic lung correlated best with oxygenation, *E*_rs_ and *E*_*L*_ (Additional file 1: 8. Table: Correlation between lung aeration and different lung parameters).

### Effect of PEEP

#### Healthy lungs

In the presence of IAH and healthy lungs, high PEEP did not affect oxygenation but increased airway plateau and esophageal pressure, and decreased *E*_*rs*_ due to decreased *E*_*L*_ (Table 1, Additional file 1: 9. Figure: Effect of PEEP on *P*/*F* ratio). Furthermore, blood pressure and cardiac output decreased (Additional file 1: 10. Table: Effect of PEEP on cardio-respiratory variables). In addition, gas volumes increased, but tissue mass was not affected. The overall proportion of normally aerated lungs increased due to a decrease in poorly aerated and atelectatic lung, with a parallel increase in the overall proportion of overdistended lung (Fig. 1).

**Fig. 2:**
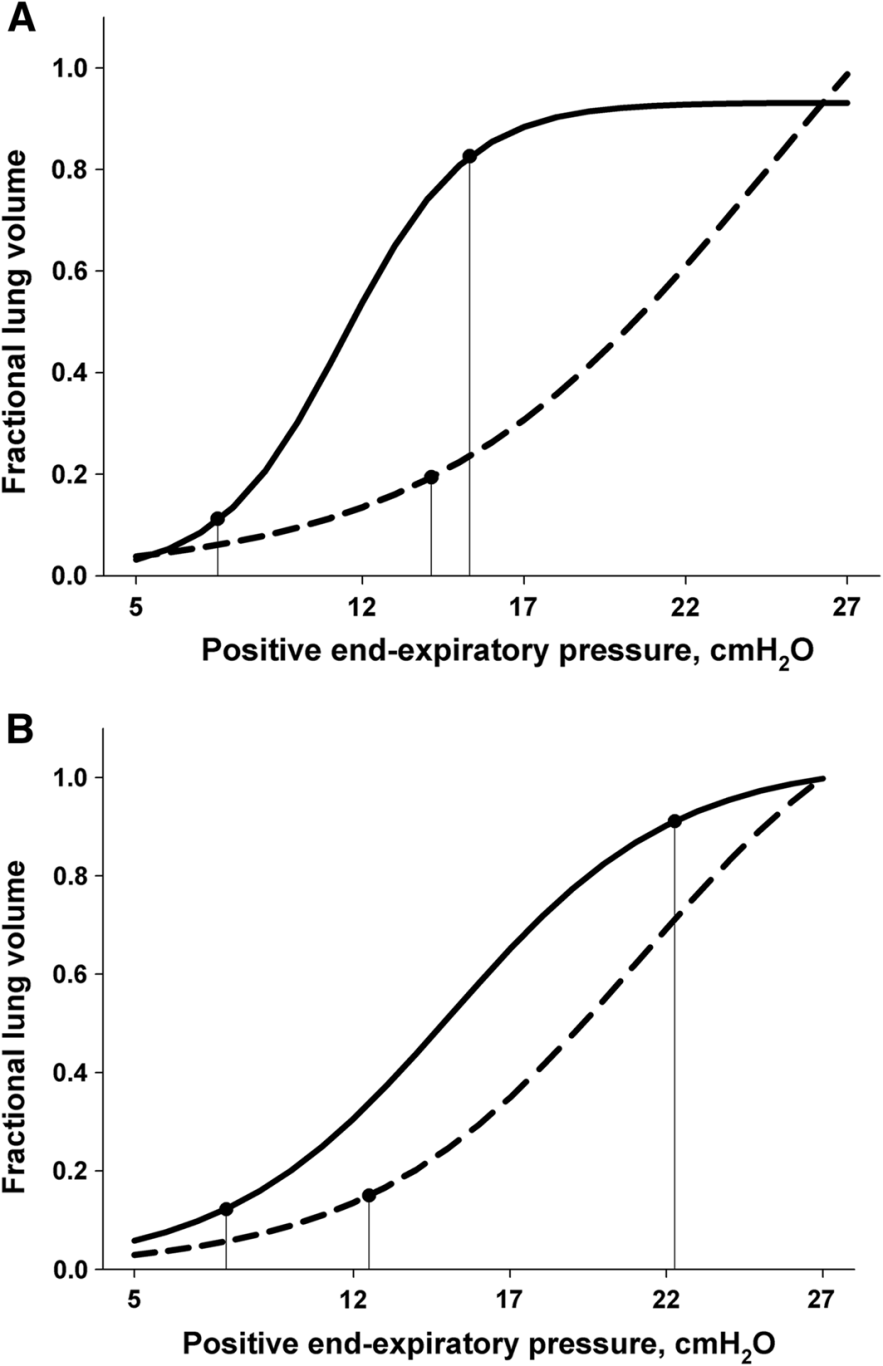
End-expiratory pressure–volume curves for atelectatic (solid line) and overdistended lung volume (dashed line) are depicted for healthy (**a**) and injured lungs (**b**). Fractional lung volumes are given as the difference between 5 and 27 cmH_2_O of positive end-expiratory pressure (PEEP). Median pressure–volume curves were derived, and lower and upper inflection points were calculated using the Venegas equation [30]. For atelectatic lung, lower and upper inflection points and for overdistended lung, lower inflection points are provided

The Venegas equation best described the PEEP—CT volume data sets when compared with the exponential and linear equations (smallest root mean square) (Additional file 1: 11. Table: Tested equations to fit pressure–volume data). Figure 2 shows end-expiratory pressure–volume curves for atelectatic and overdistended lung. Table 2 presents optimum PEEP levels based on different titration targets. In healthy lungs, the “optimal CT inflation PEEP range” was 7.5 to 14.9 cmH_2_O.

**Table 2:** Optimum PEEP relative to titration targets

PEEP titration targets	Healthy lungs PEEP, cmH_2_O	Injured lungs PEEP, cmH_2_O	*p*
*P*/*F* ratio, highest	12 (12, 27)	22 (22, 22)	0.416
*E*_rs,_, lowest	22 (22, 22)	22 (22, 22)	0.317
*E*_*W*_, lowest	22 (17, 22)	22 (17, 22)	0.854
*E*_*L*_, lowest	22 (22, 22)	22 (17, 22)	0.180
C.O., highest	5 (5, 5)	5 (5, 5)	0.317
Lung gas volume, highest	27 (27, 27)	27 (27, 27)	1.000
Overdistended lung, least	5 (5, 5)	5 (5, 5)	1.000
Normally aerated lung, most	27 (27, 27)	27 (27, 27)	1.000
Atelectatic lung, least	27 (27, 27)	27 (27, 27)	1.000
**Lower inflection point**			
Lung gas volume	8.9 (8.6, 9.4)	9.5 (9.2, 10.4)	0.138
Overdistended lung	**14.9** (14.1, 14.9)	**13.3** (12.5,14.6)	0.345
Normally aerated lung	8.3 (7.9, 8.4)	9.2 (9.1, 9.4)	**0.043**
Atelectatic lung	**7.5** (6.6, 7.5)	**8.1** (7.9,8.3)	0.345
**Upper inflection point**			
Lung gas volume	29.0 (28.4, 29.1)	28.6 (27.5, 29.8)	0.500
Overdistended lung	34.8 (34.5, 36.4)	29.7 (24, 31.8)	0.225
Normally aerated lung	27.1 (26.0, 28.4)	29.0 (28.2, 29.4)	**0.043**
Atelectatic lung	15.1 (15.0, 15.3)	22.3 (21.3, 22.4)	**0.043**

Bold indicates significant p-values or lower inflection points of atelectatic and overdistended lung with the difference defining an “optimal CT inflation PEEP range”

Median (IQR) are shown

*PEEP* positive end-expiratory pressure; *P/F ratio* arterial oxygen tension/fractional inspiratory concentration of oxygen; *E* elastance*;*_*rs*_ respiratory system; _*W*_ chest wall; _*L*_ lung; *C.O.* cardiac output

#### Injured lungs

In the presence of IAH and injured lungs, increased PEEP improved oxygenation (Table 1, Additional file 1: Effect of PEEP on *P*/*F* ratio). The effect of PEEP on plateau airway, end-expiratory, and end-inspiratory esophageal pressures, *E*_rs_ and *E*_*L*_ as well as gas volumes and the overall and segmental aeration compartments (overdistended, normally aerated, poorly aerated and atelectatic lung) paralleled those found in healthy lungs (Fig. 1, Table 1). In contrast, PEEP increased tissue mass in the presence of IAH and injured lungs. The “optimal CT distension PEEP range” was 8.1 to 13.3 cmH_2_O.

## Discussion

Optimal ventilation management in patients with IAH is still debated [7, 13, 14, 19]. This animal experiment uniquely explored the effect of different PEEP levels on the degree of atelectasis and overdistension using CT in a porcine model of IAH and lung injury. To the best of our knowledge, no other study has assessed the effect of different PEEP levels on CT measured segmental lung aeration in a porcine model of IAH and lung injury.

### Effect of IAH and oleic acid: affirmation of our animal model

Overall, this study reproduced the known negative effects of IAH (via cranial diaphragmatic shift) [12] on oxygenation, lung volume and lung mechanics in healthy and injured pig lungs: IAH reduced oxygenation mainly in injured lungs and respiratory system compliance was reduced due to a decrease in chest wall compliance [6, 8, 11–13, 21, 32, 33]. IAH reduced lung volumes and the proportion of normally aerated lung while increasing atelectasis formation [6, 34]. These findings are consistent with the literature and provide some validity to the animal model used in this animal experiment.

To create lung injury, we used oleic acid, which is directly toxic to endothelial cells causing a varying degree of interstitial and alveolar edema, hemorrhagic infiltration and fibrin deposition [35]. Our finding that oleic acid reduced gas volume without affecting tissue mass suggests that alveolar collapse and not lung edema predominated in our animal model [36]. In contrast, other investigators describe IAH to increase lung tissue mass following oleic acid [6, 11, 37] and may reflect different experimental protocols used regarding fluid management and the degree of lung injury.

### Overall respiratory effect of PEEP in the presence of IAH

In line with previous work, higher PEEP levels in this project partially reversed the above changes induced by IAH: PEEP improved lung volumes and lung compliance in both healthy and injured lungs and improved oxygenation in injured lungs [8, 11, 13, 20, 21, 33].

Surprisingly, higher PEEP levels increased lung tissue mass in injured but not in healthy lungs, suggesting that PEEP increased lung edema in the presence of IAH and lung injury [36]. The increase in lung edema in this setting can be explained by the resulting higher intra-thoracic pressures for a given tidal volume, which may itself decrease thoracic lymph drainage [38] or increase intra-thoracic blood volume [19]. Animal studies are conflicting regarding the reported effect of PEEP on lung edema [14, 24, 39]. Thus, whether higher PEEP in patients with IAH and lung injury increases the risk of lung edema remains debatable.

We found the proportion of atelectatic lung to correlate best with oxygenation, *E*_rs_ and *E*_*L*_ and suggests that oxygenation improved due to a reduction in atelectasis. In contrast to poorly aerated lung, atelectatic lung represents a shunt region that does not participate in gas exchange [25]. Muders et al. [14] found that higher PEEP levels decreased shunt perfusion and dead space ventilation in a porcine model of IAH lung injury.

### Effect of PEEP on segmental lung aeration in the presence of IAH

We found higher PEEP levels increased the overall proportion of normally aerated lungs due to a decrease in poorly aerated and atelectatic lung, mainly in the dorsal dependent lung segments. However, higher PEEP levels also increased the proportion of overdistended lung in the ventral non-dependent lung segments.

We defined the “optimal CT inflation PEEP range” as the difference between the lower inflection points of the atelectatic and overdistended lung. In theory, maximal alveolar recruitment with minimal alveolar overdistension occurs within this range [25, 30]. We found the “optimal CT inflation PEEP range” in the presence of IAH to be as low as 8 to 15 cmH_2_O and 8 to 13 cmH_2_O in healthy and sick lungs, respectively. In contrast, we found the “best PEEP”, defined as causing the smallest lung elastance [13], with 22 cmH_2_O (healthy and injured lungs) to be higher than the “optimal CT inflation PEEP range”. Our results suggest that titrating a PEEP to respiratory mechanics or oxygenation in the presence of IAH is associated with increased alveolar overdistension, which itself increases the risk of ventilator-induced lung injury [18, 22, 24, 40].

At first sight, our “best PEEP” with 22 cmH_2_O appears high but is comparable to works of others. Keenan et al. investigated different PEEP levels (5 to 20 cmH_2_O) in a porcine model of IAH and lung injury (IAP of 20 cmH_2_O) [13]. They concluded that the “best PEEP” was higher in the presence of IAH but was not influenced by the supine or prone position. Importantly, in the presence of IAH, their “best PEEP” of 17 cmH_2_O equated to 85% of IAP, which was comparable to what we found (22/27 cmH_2_O, 81%). In addition, Muders et al. [14] found a PEEP of 22 cmH_2_O to cause the least tidal recruitment on electric impedance tomography in the setting of IAP 20 cmH_2_O and lung injury.

### Clinical consequences

Our findings, that titrating PEEP to the best respiratory or oxygenation was associated with increased alveolar overdistension, should be considered cautiously. Titrating PEEP based on CT measured lung aeration and applying Venegas formula to assess overdistension lower inflection point is not practical at the beside. The proportion of overdistension, depending on PEEP, was only 1 to 5% in our study. In addition, we did not measure systemic inflammation or assess histological changes to provide a sense of harm caused by increases in overdistension.

Our model included a relatively modest degree of lung injury. However, the application of higher PEEP levels in patients with mild ARDS is not recommended [41, 42]. As the studies assessing different PEEP levels in patients in ARDS do not account for the presence or absence of IAH, the optimal PEEP to be applied in patients with IAH remains unknown [42].

As IAH and PEEP only minimally affect oxygenation in healthy lungs, the role of PEEP is to provide safe protective lung ventilation and not to improve oxygenation [19].

It remains debatable, whether patients with IAH in general may benefit from higher PEEP levels to reduce the risk of atelectotrauma and ventilator-induced lung injury remains unknown [3, 19, 22]. Experimental and human data suggest that the degree of alveolar overdistension may be a more significant contributor to the release of pro-inflammatory cytokines than the cyclic nature of the ventilatory pattern [24, 40].

### Study limitations

This study has several limitations. First, an animal experiment may incompletely represent lung injury and IAH found in critically ill patients. Second, we included a relatively small sample size, and one pig died mid-experiment. Third, we did not perform any histology or assess systemic inflammation changes associated with increased overdistension. Fourth, our experiment was performed in the presence of only one relatively high IAP level, we applied only a relatively modest degree of lung injury, and our experiment was not randomized. Fifth, examining more than five PEEP levels may have yielded physiologically more accurate Venegas equations. However, our findings of increased PEEP on bedside cardio-respiratory parameters are consistent with the literature in healthy [8, 19, 43] and in injured lungs [8, 11, 13, 20, 43, 44], providing some confidence in the validity of our experimental model.

## Conclusions

In this animal model, PEEP in the presence of IAH variably decreased the proportion of poorly aerated and atelectatic lung while increasing the proportion of normally aerated and overdistended lung in both healthy and in injured lungs. Our results suggest that titrating a PEEP to respiratory mechanics or oxygenation in the presence of IAH was associated with increased alveolar overdistension.

## Supplementary Information

**Additional file 1.** Online Supplement.

## Data Availability

The data sets used and/or analyzed during the current study available from the corresponding author on reasonable request.

## Abbreviations

CT: Computed tomography
*E*_rs_: Elastance of the respiratory system
*E*_*W*_: Elastance of the chest wall
*E*_*L*_: Elastance of the lung
IAH: Intra-abdominal hypertension
IAP: Intra-abdominal pressure
PEEP: Positive end-expiratory pressures
*P*/*F*_ratio_: Arterial oxygen tension in mmHg over fractional inspiratory oxygen concentration
