# Supplementary material for: Computed tomographic assessment of lung aeration at different positive end-expiratory pressures in a porcine model of intra-abdominal hypertension and lung injury
Source: Intensive Care Med Exp. 2021 Oct 4;9:52. doi: 10.1186/s40635-021-00416-5 (PMC8489364; doi:10.1186/s40635-021-00416-5)
Supplement: Supplementary file 1 — Additional file 1. Online Supplement. [file 40635_2021_416_MOESM1_ESM.docx]

## online supplement 1 - Methods unabriged

The study conformed to the regulations of the Australian Code for the care and use of animals for scientific purposes (1) and was approved by the Animal Ethics Committees of Murdoch University (R2588/13) and of the University of Western Australia (RA/3/900/77). Studies were performed at Murdoch University Veterinary Hospital.

### Preparation of animals, anesthesia, and ventilation

Five female pigs (Large White x Landrace x Duroc breed) with a median (IQR) weight of 29.3 (29.0 to 30.6) kg were included in this study.

Anesthesia was induced with a combination of intramuscular zolazepam, tiletamine and xylazine (2 mg/kg each) (Zoletil ®, Virbac, Milperra NSW, Australia and Xylazil 100, Ilium, Troy Laboratories, Glendenning NSW, Australia). The trachea was intubated and anesthesia was maintained with total intravenous anesthesia: thiopentone 9 mg/kg/h (Thiopentone, Troy Laboratories, Glendenning NSW, Australia); 15 mg/kg, morphine 0.1 to 0.2 mg/kg/h, and ketamine 0.4 to 0.8 mg/kg/h. One gram of intravenous vancomycin (Hospira, Melbourne VIC, Australia) was administered over 30 min. Pancuronium bromide (AstraZeneca, North Ryde NSW, Australia) was administered as a neuromuscular blocking agent with an intravenous bolus (0.1 mg/kg) followed by an intravenous infusion (0.1 mg/kg/h). The adequacy of paralysis was assessed with a peripheral nerve stimulator and a train of four stimulus pattern.

The pigs were mechanically ventilated (Babylog VN500, Draeger, Lübeck, Germany) using the following settings: volume guaranteed pressure-controlled continuous mandatory ventilation (PC-CMV/VG), F_i_O_2_ 0.6, inspiration time adjusted to obtain inspiration to expiration ratio = 1:1.5, inspiratory flow 30 L/min, tidal volume 8 mL/kg. The initial PEEP setting was 5 cmH_2_O and altered according to the experimental protocol (see below). The maximal airway pressure alarm was set above inspiratory airway pressure throughout the experiment to allow full delivery of the set tidal volume. The initial respiratory rate was adjusted to maintain an end-tidal CO_2_ of 35 - 45 mmHg. Subsequently, PEEP was the only ventilation setting altered throughout the remainder of the protocol. The cuff pressure was adjusted to 5 cmH_2_O above inspiratory pressure to minimize gas leak.

The pigs were euthanized by intravenous injection of pentobarbitone (160 mg/kg, 325 mg/mL Lethabarb, Jurox, Rutherford, NSW) at the end of the experiment.

### Lung injury

The experimental protocol was carried out first with healthy lungs, after which lung injury was created by injecting oleic acid (≥ 99 %, Sigma-Aldrich, Steinheim, Germany) into the internal jugular vein (3): an initial bolus of 0.04 mL/kg oleic acid was followed by a further bolus of 0.01 mL/kg every 10 min until a P/F ratio of <300 mmHg was established (see Online supplement 2 – Figure: Experimental process). Mild as opposed to moderate or severe lung injury was chosen due to safety concerns in the setting of IAH.

### Respiratory mechanics and lung volumes

Esophageal pressure was recorded using a thin-walled latex balloon (10 cm long) sealed over one end of a polyethylene catheter (Cardinal Health, Hoechberg, Germany) connected to a pressure transducer. Following gastric insertion, the catheter was retracted stepwise until the optimal position in the esophagus was confirmed from the pressure trace and ultimately using CT-guidance.

Airway pressure was transduced at the proximal end of the endotracheal tube. End-inspiratory (_EI_) and end-expiratory (_EE_) pressures were obtained after a pause of 3 seconds. The static elastances of the respiratory system (*E*_rs_), chest wall (*E_W_*) and lung (*E*_L_) and the transpulmonary pressures were derived as described previously (2, 3).

Arterial oxygen tension, oxygen saturation, carbon dioxide tension, hemoglobin concentration, mixed venous oxygen tension, and oxygen saturation were measured with a blood gas analyzer immediately following blood collection (Rapidlab 1200, Siemens, Leverkusen, Germany). PaO_2_ over fractional inspiratory oxygen concentration (P/F ratio), shunt and dead-space fraction were calculated using standard formulae (3).

### Hemodynamic parameters

The animals remained supine throughout the study. Mean arterial blood pressure was measured at the femoral artery. Cardiac output was measured by transpulmonary thermodilution (PiCCO, Pulsion Medical System, Feldkirchen, Germany) with ice-cold saline injected into the internal jugular vein (4). All hemodynamic pressures and IAP were zeroed at the mid-axillary line at the level of the sternum and measured during end-expiration (5). Pressure was sampled and stored continuously using Powerlab and LabChart (v7.0; ADI Instruments, Bella Vista, Australia). Data were analyzed *post-hoc* in LabChart.

Pigs were stabilized hemodynamically with 4 % succinylated gelatin solution (500 mL over the first 30 min followed by 1 mL/kg/h, Gelofusine®, B. Braun, Bella Vista NSW, Australia). Noradrenalin infusion (3 mg/50 mL) was administered if required to maintain a mean arterial pressure ≥ 70 mmHg.

### Intra-abdominal pressure generation and measurement

A large bore orogastric tube was inserted to allow continuous gastric drainage. IAP of 27 cmH_2_O (20 mmHg) was created by the insufflation of air into the peritoneal cavity through an air-tight catheter. IAP ≥ 27 cmH_2_O represents ≥ grade III IAH with is found in around 8% of patients {Kirkpatrick et al., 2013, #36870}{Reintam Blaser et al., 2019, #83873}{Regli et al., 2021, #132799}. A three-way tap connected to a transducer allowed direct measurement of IAP.

### Experimental protocol

Measurements, including a CT scan, were performed initially at baseline IAP (abdomen not inflated) and again after peritoneal inflation of air to an IAP of 27 cmH_2_O (20 mmHg). The initial PEEP level was set at 5 cmH_2_O. PEEP was incremented subsequently to 12, 17, 22, and 27 cmH_2_O (“ascending”). These PEEP levels corresponded to 19, 44, 63, 81, and 100 % of IAP, respectively. PEEP levels were then decreased (“descending”) to ascertain an optimal “deflation” PEEP. Hysteresis was assessed by comparing measurements obtained at ascending and descending PEEP levels. Recruitment maneuvers were not used. CT images and physiological measurements (see below) were obtained five minutes after a stabilization period; physiological measurements at each PEEP level and CT measurements (see below) only at descending PEEP levels. Ventilation settings were kept constant except for PEEP.

### Computed tomography image acquisition and analysis

A whole-lung helical CT scan (Siemens Somatom Emotion 16, Erlangen, Germany) was performed during an inspiratory pause followed by a repeat scan during an expiratory pause (each about 20 seconds) (6). The scan parameters were standardized to 130 kV, 110 effective mA, and 1.0 pitch. All scan acquisitions were reconstructed using 3 mm slice thickness, with a 3 mm slice interval, and a lung kernel (B90s) at each tested PEEP level.

Image analyses was performed with Maluna® software (MALUNA 3.17, Peter Herrmann, Department of Anesthesiology, Emergency and Intensive Care Medicine, University of Göttingen, Göttingen, Germany). Lungs images were manually outlined and big vessels and airways were excluded in a blinded fashion. Lung volumes were calculated and three segments along the dorso-ventral axis were performed automatically. Left and right lungs were analyzed separately, but combined in graphs and tables. Based on the degree of aeration/density of lung tissue, four aeration compartments were computed: overdistended tissue (-1,000 to -901 Hounsfield units [HU]), normally aerated tissue (-900 to -501 HU), poorly aerated tissue (-500 to -101 HU), and non-aerated (atelectatic) tissue (-100 to 200 HU) (7). Tissue mass was calculated as previously described (8).

To assess the effect of PEEP, CT lung volumes as a fraction of the difference between 5 and 27 cmH_2_O PEEP were analyzed further in Excel (v 16 for Mac, Microsoft, Redmond, Washington, USA) (9).

Three different equations were applied to the data as outlined below.

1. The Venegas equation (V = a + [b/(1+e ^–(P-c)/d^)]) describes the characteristic sigmoid shape of pulmonary (10) and other P-V curves (11). In the tested situation, V represented CT measured fractional lung volume, P represented PEEP, and a, b, c, and d represented fitting parameters. In the original Venegas equation, a and b approximate the residual volume (lower asymptote volume) and the vital capacity (upper asymptote volume) respectively (10). The upper and lower inflections points were defined as P=c+/-2d (10).
2. An exponential equation, V = e + f *Ln (P-g) where V represented CT measured fractional lung volume, P represented PEEP, and e, f, g represented fitting parameters.
3. A linear equation, V= h + i * P, where V represented CT measured fractional lung volume, P represented PEEP, and h and i represented fitting parameters.

The accuracy of each equation was assessed for describing the effect of different PEEP levels on CT volumes. Fitting parameters that best described the measured pressure-volume curve were found for each corresponding pressure-volume data set using the Excel “Solver” function. The best fit was defined as a curve resulting in the smallest root mean square between the measured and calculated pressure-volume points. Median fitting parameters were used to construct pressure-volume curves.

### Statistics

A linear mixed model was applied to assess the effect of factors (IAH, lung injury, ascending vs descending PEEP) and covariates (PEEP) on different variables using SPSS (v25, IBM, St Leonards NSW, Australia). This approach accounted for the correlation between the repeated measures on each pig. Main effect was used for analysis of respiratory and hemodynamic outcomes. Main effect plus an interaction with lung segments (PEEP and lung injury) was used for CT measured lung volumes. Laterality (left/right) was included as a fixed factor in the linear mixed model. Differences between pigs were accounted for as a random effect. Missing values were imputed based on the average relative differences between any pig with missing data and the other animals. Wilcoxon-Signed Rank test was used to compare optimum PEEP levels between healthy and injured lung. Linear regression was performed to assess for correlations. A p-value of <0.05 was considered statistically significant. For descriptive statistics, median (IQR) is reported.

### References

1. National Health and Medical Research Council (Australia), Australian Research Council, Universities Australia, CSIRO (Australia). Australian code for the care and use of animals for scientific purposes. National Health and Medical Research Council National Health and Medical Research Council, Universities Australia, CSIRO; 2013:8th Edition.

2. Regli A, Chakera J, De Keulenaer BL, Roberts B, Noffsinger B, Singh B, van Heerden PV. Matching positive end-expiratory pressure to intra-abdominal pressure prevents end-expiratory lung volume decline in a pig model of intra-abdominal hypertension. Crit Care Med. 2012;40:1879-1886.

3. Regli A, Mahendran R, Fysh ET, Roberts B, Noffsinger B, De Keulenaer BL, Singh B, van Heerden PV. Matching positive end-expiratory pressure to intra-abdominal pressure improves oxygenation in a porcine sick lung model of intra-abdominal hypertension. Crit Care. 2012;16:R208.

4. Sakka SG, Reinhart K, Meier-Hellmann A. Comparison of pulmonary artery and arterial thermodilution cardiac output in critically ill patients. Intensive Care Med. 1999;25:843-846.

5. Kirkpatrick AW, Roberts DJ, De Waele J, Jaeschke R, Malbrain ML, De Keulenaer B, Duchesne J, Bjorck M, Leppaniemi A, Ejike JC, Sugrue M, Cheatham M, Ivatury R, Ball CG, Reintam Blaser A, Regli A, Balogh ZJ, D’Amours S, Debergh D, Kaplan M, Kimball E, Olvera C. Intra-abdominal hypertension and the abdominal compartment syndrome: updated consensus definitions and clinical practice guidelines from the World Society of the Abdominal Compartment Syndrome. Intensive Care Med. 2013;39:1190-1206.

6. Gattinoni L, Caironi P, Cressoni M, Chiumello D, Ranieri VM, Quintel M, Russo S, Patroniti N, Cornejo R, Bugedo G. Lung recruitment in patients with the acute respiratory distress syndrome. N Engl J Med. 2006;354:1775-1786.

7. Grasso S, Terragni P, Mascia L, Fanelli V, Quintel M, Herrmann P, Hedenstierna G, Slutsky AS, Ranieri VM. Airway pressure-time curve profile (stress index) detects tidal recruitment/hyperinflation in experimental acute lung injury. Crit Care Med. 2004;32:1018-1027.

8. Quintel M, Pelosi P, Caironi P, Meinhardt JP, Luecke T, Herrmann P, Taccone P, Rylander C, Valenza F, Carlesso E, Gattinoni L. An increase of abdominal pressure increases pulmonary edema in oleic acid-induced lung injury. Am J Respir Crit Care Med. 2004;169:534-541.

9. Gattinoni L, Caironi P, Pelosi P, Goodman LR. What has computed tomography taught us about the acute respiratory distress syndrome? Am J Respir Crit Care Med. 2001;164:1701-1711.

10. Venegas JG, Harris RS, Simon BA. A comprehensive equation for the pulmonary pressure-volume curve. Journal of Applied Physiology. 1998;84:389-395.

11. Regli A, De Keulenaer BL, Singh B, Hockings LE, Noffsinger B, van Heerden PV. The respiratory pressure-abdominal volume curve in a porcine model. Intensive Care Med Exp. 2017;5:11.

**Online supplement 2 – Figure: Experimental process**

**Online supplement 3 – Figure: P/F ratio at different experimental conditions**


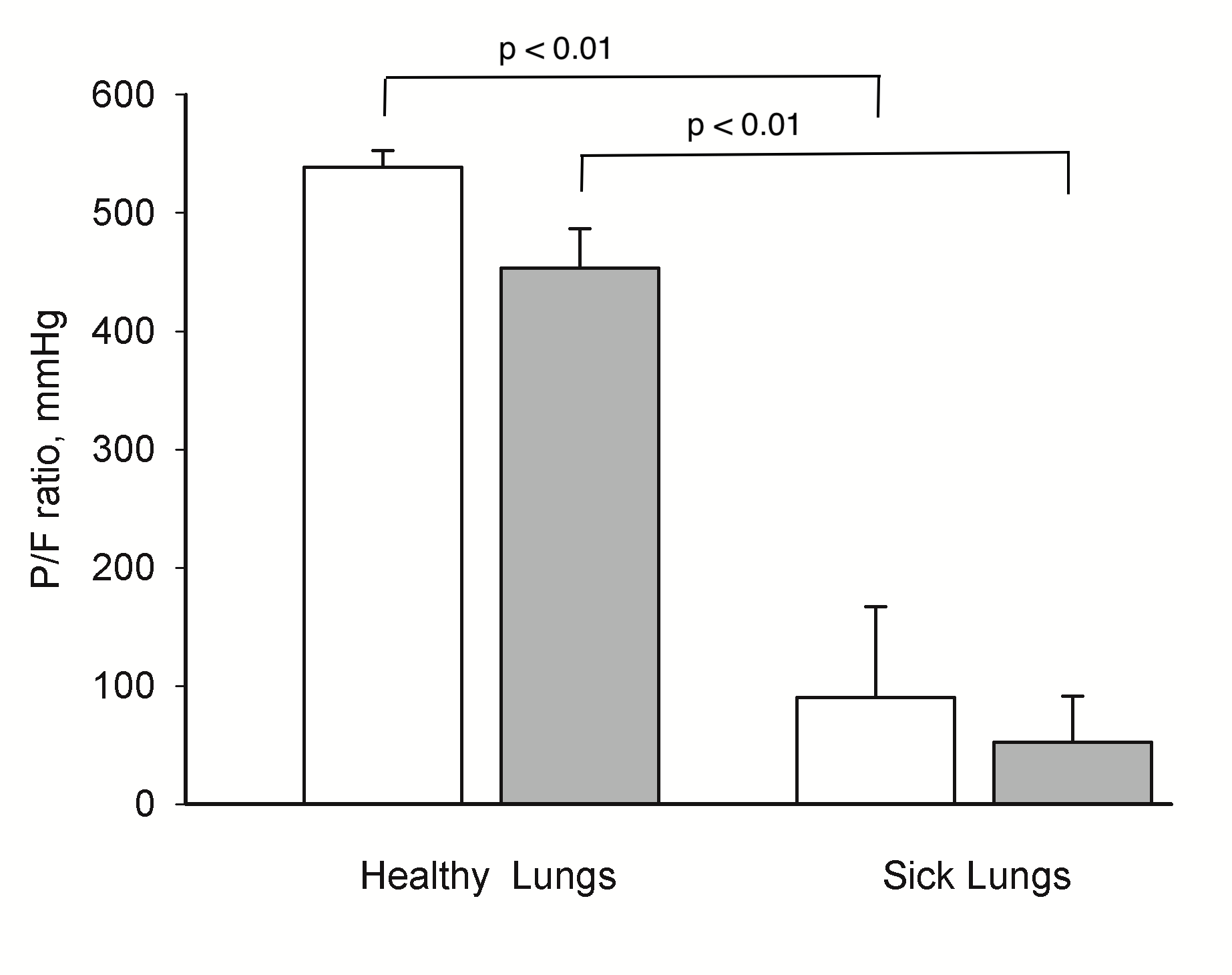


Arterial oxygen tension/fractional inspiratory concentration of oxygen (P/F ratio) in mmHg before (Healthy Lungs) and after oleic acid was given (lung injury). White and grey bars represent before (baseline) and after abdominal inflation (intra-abdominal hypertension) respectively. Mean and SE are shown. Mixed linear model was applied. Overall intra-abdominal hypertension (p=0.01) and oleic acid (p <0.01) decreased oxygenation. Only oleic acid decreased oxygenation (p<0.01) when comparing the different settings.

**Online supplement 4 - Table: Effect of PEEP on inspiratory lung aeration**

| **IAP, cmH_2_O** | **BL** | **27** | **27** | **27** | **27** | **27** |  |
| --- | --- | --- | --- | --- | --- | --- | --- |
| **PEEP, cmH_2_O (% of IAP)** | **5** | **5 (19)** | **12 (44)** | **17 (63)** | **22 (81)** | **27 (100)** | **P, PEEP** |
| **Healthy lungs** |  |  |  |  |  |  |  |
| Total lung volume, L | 1.6 (1.5,1.6) | 1.0 (1.0,1.1)# | 1.1 (1.1,1.2) | 1.3 (1.2,1.3) | 1.3 (1.3,1.4) | 1.4 (1.4,1.5) | **<0.01** |
| Lung gas volume, mL | 1175 (1111,1208) | 677 (618,694)# | 776 (760,794) | 887 (813,956) | 1013 (952,1016) | 1088 (1087,1147) | <0.01 |
| Lung tissue mass, g | 373 (343,395) | 336 (318,337) | 347 (333,352) | 355 (339,381) | 343 (322,357) | 335 (318,353) | 0.90 |
| Overdistended, % | 6 (4,7) | 3 (2,4) | 4 (3,5) | 4 (4,7) | 8 (6,10) | 10 (8,12) | **<0.01** |
| Normally aerated, % | 89 (88,90) | 77 (71,80)# | 79 (79,83) | 80 (78,81) | 82 (81,85) | 83 (80,84) | 0.16 |
| Poorly aerated, % | 5 (4,5) | 14 (12,19)# | 13 (10,15) | 12 (9,13) | 7 (6,8) | 6 (5,7) | **<0.01** |
| Atelectatic, % | 1 (1,2) | 5 (4,6) | 3 (2,3) | 2 (2,3) | 2 (1,2) | 1 (1,2) | **<0.01** |
| **Injured lungs** |  |  |  |  |  |  |  |
| Total lung volume, L | 1.3 (1.3,1.3) | 1.4 (1.1,1.5) | 1.5 (1.2,1.6) | 1.7 (1.3,1.8) | 1.8 (1.4,1.9) | 2.0 (1.5,2.0) | **<0.01** |
| Lung gas volume, mL | 893 (838,899)‡ | 802 (673,831) | 926 (713,946) | 1103 (805,1122) | 1261 (951,1285) | 1406 (1048,1431) | **<0.01** |
| Lung tissue mass, g | 394 381,405) | 454 (365462) | 496 (391,529) | 528 (393,535) | 546 (403,546) | 552 (418,552) | **<0.01** |
| Overdistended, % | 8 (5,8) | 4 (4,5) | 5 (3,5) | 6 (5,7) | 8 (6,10) | 9 (8,11) | **<0.01** |
| Normally aerated, % | 73 (67,76)‡ | 65 (58,71)‡ | 68 (62,72) | 71 (65,75) | 74 (69,78) | 76 (73,81) | **<0.01** |
| Poorly aerated, % | 14 (12,16)‡ | 13 (12,16) | 15 (14,19) | 15 (13,18) | 14 (12,17) | 11 (9,14) | **<0.01** |
| Atelectatic, % | 6 (5,7) | 15 (9,21)#‡ | 11 (6,15) | 6 (4,9) | 3 (2,4) | 2 (1,3) | **<0.01** |

BL indicates baseline intra-abdominal pressure (IAP). Otherwise IAP of 27 cmH_2_O (20 mmHg) was applied. PEEP, positive end-expiratory pressure (PEEP). Median (IQR) are given. Mixed linear effects model was used for statistical analysis. # p<0.05 between before (BL) and after IAH, ‡ p<0.5 between healthy and injured lungs.

**Online supplement 5 - Table:** Effect of IAH and lung injury on segmental lung aeration.

|  | Healthy / No IAH | | | Healthy / IAH | | | Injured / No IAH | | | Injured / IAH | | | Ventral, p values | | | | Dorsal, p values | | | |
| --- | --- | --- | --- | --- | --- | --- | --- | --- | --- | --- | --- | --- | --- | --- | --- | --- | --- | --- | --- | --- |
|  | V_1_ | D_1_ | p | V_2_ | D_2_ | p | V_3_ | D_3_ | p | V_4_ | D_4_ | p | 1v2 | 3v4 | 1v3 | 2v4 | 1v2 | 3v4 | 1v3 | 2v4 |
| Segmental volume, mL | 236 | 443 | **<0.01** | 133 | 318 | **<0.01** | 205 | 393 | **<0.01** | 165 | 410 | **<0.01** | **0.01** | 0.3 | 0.3 | 0.6 | **<0.01** | 0.7 | 0.2 | **0.04** |
| Gas volume, mL | 182 | 247 | **0.05** | 97 | 117 | 0.5 | 160 | 147 | 0.6 | 121 | 79 | 0.6 | **<0.01** | 0.2 | 0.3 | 0.6 | **<0.01** | 0.2 | **<0.01** | 0.5 |
| Tissue mass, g | 53 | 189 | **<0.01** | 33 | 176 | **<0.01** | 43 | 222 | **<0.01** | 41 | 222 | **<0.01** | 0.3 | 0.9 | 0.5 | 0.8 | 0.2 | 0.9 | 0.4 | **0.03** |
| Overdistended, % | 7.2 | 0.3 | **<0.01** | 6.4 | 0.1 | **<0.01** | 11.0 | 0.2 | **<0.01** | 8.9 | 0.1 | **<0.01** | 0.2 | **<0.01** | **<0.01** | **<0.01** | 0.9 | 1.0 | 0.9 | 1.0 |
| Normally aerated, % | 89 | 71 | **<0.01** | 83 | 33 | **<0.01** | 84 | 36 | **<0.01** | 81 | 15 | **<0.01** | 0.1 | 0.3 | 0.2 | 0.5 | **<0.01** | **<0.01** | **<0.01** | **<0.01** |
| Poorly aerated, % | 3.3 | 26 | **<0.01** | 7.4 | 50 | **<0.01** | 3.9 | 44 | **<0.01** | 5.7 | 41 | **<0.01** | 0.1 | 0.4 | 0.8 | 0.6 | **<0.01** | 0.5 | **<0.01** | **<0.01** |
| Atelectatic, % | 0.8 | 3.2 | 0.3 | 2.9 | 16 | **<0.01** | 1.4 | 21 | **<0.01** | 4.3 | 44 | **<0.01** | 0.5 | 0.3 | 0.9 | 0.6 | **<0.01** | **<0.01** | **<0.01** | **<0.01** |

End-expiratory values are shown. Intra-abdominal hypertension (IAH), intra-abdominal pressure (IAP) of 27 cmH_2_O (20 mmHg) was applied. Lungs were divided into ventral (V), mid, and dorsal (D) segments. Medial segment omitted to better demonstrate the effect of intra-abdominal hypertension and oleic acid. v, versus. Median segmental lung volumes in mL and mean proportion of segmental lung aeration per total lung segment volume in % are provided. Mixed linear effects model was used for statistical analysis.

**Online supplement 6 – Figure: Effect of IAH and lung injury on lung volumes**





Segmental end-expiratory lung volumes measured by computed tomography during different conditions being: healthy lungs and not inflated abdomen (baseline), healthy lungs and intra-abdominal hypertension (IAH), injured lungs and not inflated abdomen (baseline), and injured lungs and IAH. Three lung segments are depicted at each condition with left to right representing ventral, medial and dorsal lung segment respectively. Segmental lung volumes are a composite of overdistended (light grey on the top, -1,000 to -901 HU), normally aerated (dark grey, -900 to -501 HU), poorly aerated (light grey third from top, -500 to -101 HU) and non-aerated atelectatic atelectatic lung (black, -100 to 200 HU). Mean and SE are shown.

**Online supplemental 7 - Table:** Effect of PEEP on segmental lung aeration

| Lung condition | Healthy | | | | | | Injured | | | | | |
| --- | --- | --- | --- | --- | --- | --- | --- | --- | --- | --- | --- | --- |
| Region | Ventral | | | Dorsal | | | Ventral | | | Dorsal | | |
| PEEP, cmH_2_O | 5 | 27 | p | 5 | 27 | p | 5 | 27 | p | 5 | 27 | p |
| Segmental volume, mL | 133 | 252 | 1.0 | 318 | 473 | 0.6 | 165 | 306 | 0.8 | 410 | 610 | 0.1 |
| Gas volume, mL | 97 | 205 | 0.9 | 117 | 295 | **<0.01** | 121 | 243 | 0.2 | 79 | 311 | **<0.01** |
| Tissue mass, g | 33 | 45 | 0.6 | 176 | 171 | 1.0 | 41 | 60 | 0.3 | 222 | 286 | 1.0 |
| Overdistended, % | 6.4 | 18 | **<0.01** | 0.1 | 1.3 | 1.0 | 8.9 | 19 | **<0.01** | 0.1 | 0.8 | 1.0 |
| Normally aerated, % | 83 | 79 | 1.0 | 33 | 79 | **<0.01** | 81 | 77 | 1.0 | 15 | 55 | **<0.01** |
| Poorly aerated, % | 7.4 | 2.4 | 1.0 | 50 | 16 | **<0.01** | 5.7 | 3.0 | 1.0 | 41 | 38 | 1.0 |
| Atelectatic, % | 2.9 | 0.8 | 1.0 | 16 | 3.8 | **<0.01** | 4.3 | 1.1 | 1.0 | 44 | 5.8 | **<0.01** |

Intra-abdominal pressure (IAP) of 27 cmH_2_O (20 mmHg) was applied. Lungs were divided into ventral, mid, and dorsal segments. Medial segment omitted to better demonstrate the effect of positive end-expiratory pressures (PEEP) in the different end-expiratory lung segments. Median segmental lung volumes in mL and mean proportion of segmental lung aeration per total lung segment volume in % are provided. Mixed linear effects model was used for statistical analysis.

**Online supplement 8:** Correlation between lung aeration and different lung parameters

|  | **PaO_2_** | | **PaCO_2_** | | ***E*rs** | | ***E*cw** | | ***E*_L_** | |
| --- | --- | --- | --- | --- | --- | --- | --- | --- | --- | --- |
| **Lung variables** | **R^2^** | **p** | **R^2^** | **p** | **R^2^** | **p** | **R^2^** | **p** | **R^2^** | **p** |
| **Total volume** | 0.002 | 0.75 | 0.02 | 0.27 | 0.43 | 0.04 | 0.07 | 0.48 | 0.43 | 0.04 |
| **Gas volume** | 0.02 | 0.31 | 0.08 | 0.04 | 0.60 | 0.01 | 0.06 | 0.51 | 0.63 | 0.01 |
| **Tissue mass** | 0.04 | 0.16 | 0.04 | 0.16 | 0.04 | 0.57 | 0.08 | 0.42 | 0.02 | 0.72 |
| **Overdistended** | 0.07 | 0.06 | 0.00 | 0.86 | 0.05 | 0.55 | 0.02 | 0.72 | 0.04 | 0.59 |
| **Normally aerated** | 0.07 | 0.06 | 0.13 | 0.01 | 0.69 | 0.00 | 0.07 | 0.46 | 0.72 | 0.00 |
| **Poorly aerated** | 0.11 | 0.01 | 0.11 | 0.02 | 0.46 | 0.03 | 0.00 | 0.88 | 0.56 | 0.01 |
| **Atelectatic** | **0.45** | **0.00** | 0.16 | 0.00 | **0.88** | **0.00** | 0.09 | 0.41 | **0.93** | **0.00** |

Linear regression analysis to test for correlations. Only end-expiratory values are given. PaO_2_, arterial oxygen tension; PaCO_2_ , arterial carbon dioxide tension; *E*rs, respiratory system elastance; *E*_CW_, chest wall elastance; *E*_L_  lung elastance; CT; computed tomography. p value indicating significance, R^2^ indicating coefficient of determination.

**Online supplement 9 – Figure: Effect of PEEP on P/F ratio**





Arterial oxygen tension/fractional inspiratory concentration of oxygen (P/F ratio) in mmHg in function of different levels of positive end-expiratory pressures (PEEP). Abdomen was inflated to an intra-abdominal pressure of 27cmH_2_O (20mmHg). PEEP was stepwise increased (white circles, “healthy ascending”) and then decreased (black circles, “healthy descending”) in healthy lungs before creation of lung injury with oleic acid. Thereafter PEEP was increased stepwise (unfilled square symbols, “sick ascending”) and then decreased (black square symbols, “sick descending”). Mean and SE are shown. Mixed linear model was applied. Overall oleic acid (p<0.01) decreased, PEEP increased (p=0.03) oxygenation. Descending as opposed to ascending PEEP was associated with increased oxygenation only in injured lungs (p<0.01).

**Online supplement 10 – Table:** Effect of PEEP on hemodynamic variables

| **IAP, cmH_2_O** | **BL** | **27** | **27** | **27** | **27** | **27** |  |
| --- | --- | --- | --- | --- | --- | --- | --- |
| **PEEP, cmH_2_O (% of IAP)** | **5** | **5 (19)** | **12 (44)** | **17 (63)** | **22 (81)** | **27 (100)** | **P, PEEP** |
| **Healthy lungs** |  |  |  |  |  |  |  |
| C.O., L/min | 4.2 (4.1,4.5) | 4.4 (4.0,4.7) | 3.9 (3.6,3.9) | 3.2 (3.2,3.4) | 2.9 (2.8,2.9) | 2.8 (2.7,2.9) | **<0.01** |
| *P*_a,mean_, mmHg | 95 (85,101) | 106 (105,110) | 104 (93,105) | 108 (99,111) | 96 (84,100) | 100 (84,101) | **0.05** |
| HR, beats/min | 79 (78,81) | 88 (86,101) | 90 (82,97) | 93 (77,104) | 84 (79,98) | 102 (90,102 | 0.96 |
| NA, μg/kg/min | 5 (4,20) | 5 (4,20) | 5 (4,20) | 5 (4,20) | 5 (4,20) | 5 (4,20) | 0.49 |
| *P*_cv_, mmHg | 6 (5,6) | 17 (17,18) | 21 (19,22) | 22 (21,24) | 26 (23,27) | 26 (26,26) | **<0.01 *** |
| **Injured lungs** |  |  |  |  |  |  |  |
| C.O., L/min | 4.0 (3.4,4.8) | 4.2 (4.0,4.5) | 3.3 (3.0,3.7) | 3.2 (2.8,3.2) | 3.1 (3.0,3.6) | 3.3 (2.5,3.9) | **0.01** |
| *P*_a, mean_, mmHg | 117 (91,118) | 110 (84,136) | 97 (69,126) | 100 (75,126) | 90 (85,112) | 85 (76,104) | 0.13 |
| HR, beats/min | 135 (135,136) | 156 (152,190) | 153 (136,188) | 162 (132,178) | 161 (126,180) | 128 (125,130) | 0.91 |
| NA, mcg/kg/min | 83 (23,116) | 66 (20,95) | 62 (20,76) | 58 (20,114) | 50 (20,76) | 38 (20,43) | 0.95 |
| *P*_cv_, mmHg | 11 (10,11) ‡ | 16 (13,18) | 19 (16,21) | 23 (18,29) | 25 (21,29) | 26 (24,29) | **<0.01** |

Intra-abdominal pressure (IAP) of 27 cmH_2_O (20 mmHg) was applied. Positive end-expiratory pressure (PEEP) was increased stepwise (ascending) then decreased (descending). Only descending values are provided. Hysteresis, difference between ascending and descending PEEP; C.O., cardiac output; *P*_a, mean_, mean arterial pressure; HR, heart rate; NA, noradrenaline; *P*_cv_, central venous pressure. Median (IQR) are given. Mixed linear effects model was used for statistical analysis. No differences found between before (BL) and after IAH, ‡ p<0.5 between healthy and injured lungs, * p<0.05 indicating presence of hysteresis.

**Online supplement 11:** Tested equations to fit pressure-volume data

| Setting | **Venegas equation** | | | | | | | **Exponential equation** | | | | **Linear equation** | | |
| --- | --- | --- | --- | --- | --- | --- | --- | --- | --- | --- | --- | --- | --- | --- |
|  | a | b | c | d | LIP | UIP | RMS | e | f | g | RMS | h | i | RMS |
| **Healthy Lungs,**  **end-expiration** |  |  |  |  |  |  |  |  |  |  |  |  |  |  |
| Total lung volume | 0.0 | 1.2 | 18.2 | 4.9 | **8.5** | **27.9** | 0.04 | 0.0 | 26.4 | 11.3 | 0.10 | 0.00 | 0.03 | 0.48 |
| Lung gas volume | 0.0 | 1.2 | 19.0 | 5.0 | **9.0** | **29.0** | 0.05 | 0.00 | 26.5 | 11.3 | 0.09 | 0.00 | 0.03 | 0.48 |
| Overdistended | 0.0 | 1.6 | 24.7 | 5.3 | **14.1** | **35.3** | 0.03 | 0.00 | 26.8 | 8.1 | 0.04 | 0.00 | 0.03 | 0.46 |
| Normally aerated | 0.0 | 1.1 | 17.5 | 4.8 | **7.9** | **27.1** | 0.04 | 0.00 | 26.3 | 12.2 | 0.10 | 0.00 | 0.03 | 0.49 |
| Poorly aerated | 0.0 | 1.1 | 17.7 | 4.5 | **8.8** | **26.6** | 0.04 | 0.00 | 25.5 | 17.4 | 0.19 | 0.00 | 0.03 | 0.49 |
| Atelectatic | 0.0 | 0.9 | 11.4 | 1.9 | **7.5** | **15.3** | 0.06 | 0.00 | 25.5 | 17.4 | 0.20 | 0.00 | 0.04 | 0.53 |
| **Injured Lungs,**  **end-expiration** |  |  |  |  |  |  |  |  |  |  |  |  |  |  |
| Total lung volume | 0.0 | 1.2 | 19.1 | 4.5 | **10.2** | **28.1** | 0.04 | 0.00 | 26.5 | 10.8 | 0.09 | 0.00 | 0.03 | 0.48 |
| Lung gas volume | 0.0 | 1.2 | 19.0 | 4.8 | **9.5** | **28.6** | 0.03 | 0.00 | 26.5 | 10.9 | 0.09 | 0.00 | 0.03 | 0.48 |
| Overdistended | 0.0 | 1.3 | 21.1 | 4.3 | **12.5** | **29.7** | 0.02 | 0.00 | 26.6 | 8.7 | 0.07 | 0.00 | 0.03 | 0.48 |
| Normally aerated | 0.0 | 1.2 | 19.0 | 5.0 | **9.1** | **29.0** | 0.04 | 0.00 | 26.5 | 11.0 | 0.09 | 0.00 | 0.03 | 0.48 |
| Poorly aerated | 0.0 | 1.2 | 17.5 | 2.1 | **13.2** | **21.7** | 0.16 | 0.00 | 25.9 | 10.2 | 0.17 | 0.00 | 0.03 | 0.52 |
| Atelectatic | 0.0 | 1.0 | 15.1 | 3.6 | **7.9** | **22.3** | 0.04 | 0.00 | 25.7 | 13.3 | 0.15 | 0.00 | 0.04 | 0.51 |
| **Healthy Lungs,**  **end-inspiration** |  |  |  |  |  |  |  |  |  |  |  |  |  |  |
| Total lung volume | 0.0 | 1.3 | 20.4 | 4.9 | **10.6** | **30.1** | 0.05 | 0.0 | 26.6 | 11.3 | 0.08 | 0.00 | 0.03 | 0.47 |
| Lung gas volume | 0.0 | 1.2 | 19.7 | 4.8 | **10.0** | **29.4** | 0.04 | 0.0 | 26.5 | 11.0 | 0.08 | 0.00 | 0.03 | 0.47 |
| Overdistended | 0.0 | 1.5 | 23.9 | 4.8 | **14.2** | **33.6** | 0.02 | 0.0 | 26.8 | 7.8 | 0.05 | 0.00 | 0.03 | 0.47 |
| Normally aerated | 0.0 | 1.2 | 17.9 | 4.5 | **8.9** | **26.8** | 0.06 | 0.0 | 26.4 | 12.5 | 0.10 | 0.00 | 0.03 | 0.49 |
| Poorly aerated | 0.0 | 1.2 | 19.6 | 3.9 | **11.7** | **27.5** | 0.06 | 0.00 | 25.5 | 17.4 | 0.23 | 0.00 | 0.03 | 0.47 |
| Atelectatic | 0.0 | 1.0 | 13.1 | 3.7 | **5.6** | **20.6** | 0.12 | 0.00 | 25.5 | 17.4 | 0.20 | 0.00 | 0.04 | 0.53 |
| **Injured Lungs,**  **end-inspiration** |  |  |  |  |  |  |  |  |  |  |  |  |  |  |
| Total lung volume | 0.0 | 1.1 | 19.1 | 4.1 | **11.0** | **27.2** | 0.05 | 0.0 | 26.6 | 10.2 | 0.10 | 0.00 | 0.03 | 0.48 |
| Lung gas volume | 0.0 | 1.1 | 18.9 | 4.1 | **10.7** | **27.1** | 0.04 | 0.0 | 26.5 | 10.1 | 0.10 | 0.00 | 0.03 | 0.48 |
| Overdistended | 0.0 | 1.4 | 22.7 | 4.4 | **13.8** | **31.6** | 0.04 | 0.0 | 26.8 | 8.2 | 0.08 | 0.00 | 0.03 | 0.47 |
| Normally aerated | 0.0 | 1.1 | 19.1 | 4.3 | **10.6** | **27.6** | 0.04 | 0.0 | 26.6 | 10.1 | 0.09 | 0.00 | 0.03 | 0.48 |
| Poorly aerated | 0.0 | 1.2 | 13.4 | 0.4 | **12.6** | **14.2** | 0.88 | 0.0 | 22.9 | 19.3 | 1.02 | 0.15 | 0.05 | 1.04 |
| Atelectatic | 0.0 | 1.0 | 15.1 | 3.6 | **7.9** | **22.3** | 0.04 | 0.0 | 25.7 | 13.3 | 0.15 | 0.00 | 0.04 | 0.51 |

Constants and root mean square (RMS) of different equations are shown. Lung volumes as a fraction of the difference between 5 - 27 cmH_2_O of positive end-expiratory pressure (PEEP). The tested equations were: Venegas V = a + [b/(1+e –(P-c)/d)] {Venegas et al., 1998, #57981}, exponential V = e + f *Ln (P-g), and linear V= h + i * P. V represented computed tomography measured end-expiratory fractional lung volume, P represented PEEP, and a, b, c, d, e, f, g, h, and i represented fitting parameters. In the original Venegas equation, a and b approximate the residual volume (lower asymptote volume) and the vital capacity (upper asymptote volume) respectively {Venegas et al., 1998, #57981}. LIP, lower inflection point and UIP, upper inflection point are defined as P=c+/-2d {Venegas et al., 1998, #57981}.
